# Mapping the Landscape of Organized Sport in a Community: Implications for Community Development

**DOI:** 10.3389/fspor.2022.855798

**Published:** 2022-04-12

**Authors:** Alison Doherty, Swarali Patil, Justin Robar, Abby Perfetti, Kendra Squire

**Affiliations:** School of Kinesiology, Western University, London, ON, Canada

**Keywords:** community sport organizations, landscape, nonprofit, commercial, community development, ArcGIS

## Abstract

This study presents the landscape of private community sport organizations in the City of London, Ontario, Canada based on a profile of organizational features that align conceptually with critical aspects of community development. Features representing the scope—variety of sports offered, program age targets, and other offerings—and operations—nonprofit/commercial sector, open/closed program type, independent/affiliated/franchise status, and shared/exclusive facility use—of community sport organizations were captured from publicly available information about the population of 218 organizations. The location of sport delivery points for each organization was also mapped. The landscape is characterized by a balance of nonprofit and commercial organizations, offering a wide variety of sports, across all ages and City districts, but predominantly offered through closed programming that typically requires an extended financial commitment. Community sport organizations in this city are also most likely to operate independently, and share facilities. These features, and the landscape, are conceptualized as having implications for access, social inclusion, engagement and citizenship, and social capital that are fundamental to community development. Mapping the landscape in this community provides a valuable resource for understanding that potential.

## Introduction

The wealth of a community may be measured in part by the range, availability, and accessibility of organized sport programs and services for individuals and families within its geographic boundaries (cf. Spaaij, 2009). Nonprofit and commercial organizations offer programs for children, youth, adults and/or seniors, of varying abilities, that complement—and increasingly replace—public or municipal offerings. Organized sport provides opportunities for individuals to realize important physical and psychosocial benefits of participation, as well as family wellbeing through joint engagement (e.g., Spaaij, 2010; Downward et al., 2017). It can also be a basis for community development, through the practice and realization of social changes that contribute to the “betterment” of a community (Doherty and Rich, 2015). Such positive changes in the context of sport include generating social capital through relationships and networks, fostering social inclusion of marginalized groups, and promoting a sense of community identity and citizenship (Tonts, 2005; Spaaij, 2009; Doherty and Rich, 2015; Davies et al., 2019).

The contribution of community sport organizations to community development in Canada is of particular interest because the large majority of Canadians (77% of children and youth, 61% of adults) participate in sport in an organized setting [Canadian Fitness Lifestyle Research Institute [CFLRI], 2018, 2019]. The scope, operations, and location of these organizations and their programs can have important implications for sport's contribution to community development (Doherty and Rich, 2015). The profile of organizations provides a foundation for assessing emphases and gaps in the sport landscape—and wealth—of the community.

For this project, we define sport as any activity that involves training and/or competition with some level of physical intensity and organization or structure (Berrett, 2018). We focus on “community as context” (as opposed to community as an outcome) (Rich et al., 2021, p. 4) in this study, with reference to physical space in a geographic area that is labeled officially and legally as the City of London, Ontario. Thus, our project focuses on community sport organizations whose primary function is the delivery of sport within this municipality.

Our efforts align with related endeavors that ‘map’ or ‘take stock’ of a type or types of organizations in a given community or region. For example, Gronbjerg and Nelson (1998) identified the profile of small religious nonprofit service organizations in the state of Illinois, to better understand the features and variations of this “relatively invisible” sub-sector (p. 13). A valid and comprehensive picture of historically “hidden” organizations (Gronbjerg and Nelson, 1998, p. 14) provides a basis for research and policy activities that have been largely missing or at least not well-supported. In one of several projects mapping nonprofits in various American communities, Twombly et al. (2000) reported on the size, scope of activities, and location of nonprofits throughout the City of Philadelphia, in an effort to capture the “neighborhood assets” (p. iii) that provide a platform for a community building strategy. Their findings include the geospatial density of nonprofits in that city, and most common types of programs and services offered and their locations. In a community case study, Toepler (2003) compared the profiles of small and large nonprofit cultural organizations in one county with the intent of better understanding their economic dimensions as part of the grassroots base of the nonprofit sector. In the Canadian context, Elson and Hall (2012) examined the profile of social enterprises in two provinces to understand, and compare, their structure, purpose, and operational activity, and thus, better understand the sector as a whole within those regions. The resultant knowledge helps to make the case for enhanced policy support of the sector, ensuring it is on the political agenda. This body of research is largely prompted by the lack of data about small, grassroots organizations, including their scope of activity and spatial distribution. It is based on secondary data drawn from government records, primary data through organization surveys, or both.

The purpose of the current study was to describe the landscape of organized community sport in one community—London, Ontario (popn. 409,000)—as a foundation for understanding its contribution to community development. The endeavour addresses the call by Rich et al. (2021) for contextual analyses of community as context for sport, and a platform for a more critical approach to understanding the role of organized sport in positive community development (cf. Coakley, 2011). It also provides a foundation for asset-based planning and further implementation and evaluation activities that are necessary for sustained community development or change (Vail, 2007). The study addresses the need for practical research that can inform sport planning, management, and leverage for community development (Schulenkorf, 2012). It represents a start at monitoring and evaluation for future policy, strategy, and planning that relies on systematic evidence of the landscape (Schulenkorf, 2012).

The study was guided by the following research questions:

1. What is the scope, operations, and location profile of community sport organizations in London, Ontario?
2. What are the strengths, and what are the gaps, in the landscape of community sport in London, Ontario?

The focus of the study was private commercial and nonprofit sport clubs. Public or municipal sport programs and centers are a critical part of the multi-sector sport provision that characterizes communities, however they were excluded from this mapping exercise, as were unstructured sport activities, such as pickup basketball at a school or public outdoor court. While these programs and activities also have the potential to contribute to community development, we were interested specifically in exploring the community sport landscape represented by private organizations, beyond the public domain. To ensure the resultant profile would be relevant to municipal leaders, our research team consulted with representatives of the City of London Parks & Recreation unit regarding the focus and design of the project. Through several meetings, we gained insights to the City's policy and strategic planning interests, municipal planning districts for geospatial mapping, relevant organizational features to measure, and direction with sampling.

## Conceptual Framework—Sport for Community Development

To frame the paper, we outlined a conceptual model that aligns important aspects of community development and particular features of community sport organizations that may be expected to contribute to those aspects. Here we review community development and sport's contribution to that, followed by a consideration of how sport delivery organizations themselves can foster that contribution.

In the *Community Development Reader*, DeFilippis and Saegert (2012) begin by describing communities as “the realm in which social reproduction occurs [through institutions and] activities that sustain us physically, emotionally, socially, and psychologically” (p. 3). Community development, then, is about positive change with regard to economic, social, cultural or environmental circumstances that support its members (Christenson et al., 1989). It is fundamentally about members of a community helping each other by identifying and addressing common needs and interests (Vail, 2007), through the organization and provision of resources to help the community thrive (DeFilippis and Saegert, 2012). Sport and physical recreation has had considerable attention as a mechanism by which people can work, and play, collectively in the pursuit of healthy experiences—and social reproduction—within their community.

In particular, community sport has been linked with social inclusion of marginalized or underserved individuals and groups as participants and leaders through accessible, targeted opportunities for meaningful involvement (e.g., Maxwell et al., 2013; Wheaton et al., 2017; Jeanes et al., 2018). Community sport is also linked with opportunities for engagement, active citizenship, and sociopolitical development as individuals and groups become involved in the sustainability of the organization, and of sport in the community (Cousens and Barnes, 2009; Christens and Dolan, 2011; Maxwell et al., 2013; Morgan, 2013; Darcy et al., 2014; Wheaton et al., 2017). The organization and programming of community sport can shape personal and community identity or citizenship, as individuals connect with their chosen sport, fellow participants or club leaders, and the community as a whole (Misener and Mason, 2006; Skinner et al., 2008; Maxwell et al., 2013; Wheaton et al., 2017). Sport has increasingly been linked with individual and community social capital, characterized as trust and a willingness to help each other out, that may be generated through relationships and networks, both within and beyond a given sport context (Doherty and Misener, 2008; Skinner et al., 2008; Cousens and Barnes, 2009; Cousens et al., 2012; Misener and Doherty, 2012; Maxwell et al., 2013; Darcy et al., 2014; Doherty and Rich, 2015; Hill et al., 2021).

The positive change that is community development is purported to happen most effectively in a ‘bottom-up’ approach (Bolton et al., 2008) that is community-driven and constitutes individuals and groups undertaking a process of helping themselves (Pedlar, 1996), while building on existing assets (Vail, 2007). This change may be an intentional effort, as exhibited by formal community development organizations such as food banks, housing co-operatives, and neighborhood watch groups. It may also be residual, as a further outcome or extension of an organization's primary program of activities. Certainly, local sport clubs align with the notion that communities are (best) developed from the bottom-up or “building from below” (Schulenkorf, 2012, p. 3; also Doherty et al., 2014). However, “while sport has the potential to achieve community development outcomes… sport does not always do this, nor does it necessarily try” (also Doherty and Rich, 2015, p. 131; also Chalip, 2015; Schlesinger and Doherty, 2021). The contribution of organized sport to community development may not be the primary or even overt intention of community sport organizations (Vail, 2007; Edwards, 2015), but nonetheless they are believed, expected, and known to play that role. The Canadian Centre for Ethics in Sport [CCES] (2018) report on Canadians' Attitudes Towards Sport indicates that the large majority of Canadians believe community-level sport can contribute to good health (91%) and can instill character in youth (84%). Further, the top reported reasons for getting involved in organized sport include friendship and community, socialization, and teamwork (CCES, 2018).

Following from research that has taken stock of the landscape of types of organizations in particular communities (Gronbjerg and Nelson, 1998; Twombly et al., 2000; Toepler, 2003; Elson and Hall, 2012), we focused on the scope, operations, and location features of community sport organizations deemed relevant to community development. As a starting point with this study, we considered the variety of sports, program age targets, and other types of activities (e.g., social) as indicative of the scope of opportunities available in a community with implications for citizens' access, inclusion, and identity. We considered organizations' operational features of sector (nonprofit/commercial), program type (open or drop-in/closed or registration-based), independent/affiliated/franchise status, and facility use (shared/exclusive) as having implications for community-driven programming that is fundamental to community development, access to such programming, engagement with an organization, and the generation of social capital. Finally, geospatial location of program delivery across the community may be expected to have implications for access, and the inclusion, engagement, identity, and social capital which may flow from that. This conceptualization is illustrated in Table 1, with important aspects of community development aligning with organizational features that may be factors in that development. These alignments are certainly not exhaustive, and further dynamics among the aspects and features may also be relevant; for example, the opportunity to engage socially and politically through an organization may not only be dependent on volunteer opportunities available but also on the location and therefore access to the organization. We also did not measure in this project the actual degree of access, inclusion, engagement, identity, or social capital that may be associated with the organizational features. However, the resultant profile of organizations illustrates the landscape of organized sport that may be expected to have implications for community development in the focal community.

**Table 1:** Conceptual alignment of aspects of community development and features of community sport organizations.

**Aspect of community development**	**Organizational feature**
Access to programming	Sector (nonprofit/commercial): • May have implications for cost Program type (open/closed): • Implications for extended vs. one-time financial commitment Location (density of sport delivery points in a given area): • Implications for ease of access
Social inclusion in programming, the organization	Variety of sports: • Implications for meeting interests of a diverse community Program target age (children, youth, adults, seniors, all): • Implications for proportion of opportunities to meet interests Other offerings: • Typically social activities, provide an additional or alternative outlet for participation
Engagement, active citizenship, sociopolitical development	Sector (nonprofit/commercial): • Implications for volunteer opportunities
Identity (personal, community)	Variety of sports: • Implications for individual identity based on availability (and access and inclusion) of a given sport • Implications for community identity, based on predominance of a single sport, or breadth of sport offerings Other offerings: • Implications for alternative opportunities to identify with and through a sport club
Social capital	Independent/affiliated/franchise status: • Implications for community-driven organization and programming vs. top-down direction from an umbrella or parent organization Facility use (shared/exclusive): • Implications for opportunity to generate social capital (trust, cooperation, reciprocity) among organizations (leaders, participants) that share a facility

## Method, Analyses

Determining the landscape of organized community sport in London was a multi-step process that involved: (1) determining the content of the mapping instrument (organizational features to measure), (2) identifying the population of organizations that fit the criteria (community-based, sport organization serving/located within the City boundaries, web presence), (3) web-based secondary data collection, (4) geospatial mapping, and (5) data analysis and interpretation.

The instrument was developed to capture key features of community sport organizations that are relevant to describing the landscape of community sport. These were identified from a review of the literature that has considered the mission and goals, structure and design, and offerings of these institutions (Thiel and Mayer, 2009; Adams, 2011; Vos et al., 2012; May et al., 2013; Nichols et al., 2015; King and Church, 2017; Misener and Misener, 2017; Lang et al., 2019; Rossi et al., 2020; Hill et al., 2021). We accounted for different types of private community sport delivery organizations to capture their common and unique characteristics. The research literature was delimited to publications since 2010 to ensure a contemporary focus. The City of London *2019 Parks and Recreation Master Plan* was also reviewed—and City representatives were consulted—to identify features of particular importance to the City in the subsequent few years, for example active living, inclusion and access, and programming for an aging population. The City master plan also identifies the growth of high density residential communities as a consideration for recreational planning. Over 35 organizational features were drawn from the literature, ranging from mission and goals, to certification for coaches, to socially responsible initiatives (e.g., recycling). This was reduced to a final list of 29 distinct characteristics that define and distinguish community sport organizations, and were determined to be collectable from publicly available data (website, social media). A selection of these features was identified as aligning with sport's potential contribution to community development, as indicated in the conceptual framework, however the full list of features is provided in the Appendix).

The population of organizations was identified both systematically and organically. A list of possible sports was generated from research team members' own knowledge and further brainstorming, and was added to as additional sports came to light. Organizations that offer these sports in London, Ontario were identified by: (1) a search of Ontario provincial sport organizations' websites for London clubs, (2) a review of past and current issues of a community sport-focused magazine *SportsXpress* that regularly profiles local clubs in London, (3) a Google search by “London, Ontario” and a given sport, and (4) a list of facility users from the City of London. Each identified organization was checked for a website or social media presence (esp. Facebook), and to confirm that their primary focus is sport. Only organizations with an online presence (website, social media) from which data could be collected were included in the project. An initial list of 228 community sport organizations was identified, although 10 of those were discovered to no longer exist at the time of data collection or not to have a website or Facebook presence and so were excluded. A final total of 218 organizations comprised the study sample.

Document-based data collection (Patton, 2015), drawing from organization websites or social media, took place from May to August of 2020. This approach was chosen largely because the project was undertaken during the original COVID-19 lockdown in London, Ontario (starting March 2020). By public health order, all sport organizations ceased operating and it would have been very challenging to connect with organization representatives to collect or even verify information. Document-based and secondary data have been a common source of information for profiling in other mapping studies (e.g., Gronbjerg and Nelson, 1998; Twombly et al., 2000). It became clear that community sport organizations had not changed any information on their websites, with some only providing a statement that they had suspended operations until further notice. In addition, secondary data collection was consistent with the ability to collect a large amount of information in a concentrated period of time that ensured a cross-sectional view of the landscape of community sport in the City. The data set was 85–100% complete for the variables of interest in this paper. Data were checked again in the same months of 2021, when sport organizations were still in lockdown or beginning to resume activities, in order to fill in any missing data from 2020, however there were no changes to the information collected regarding scope, operations or location of the organizations. Thus, the profile represents community sport organizations and their programs pre-COVID. This provides a valuable basis for understanding the landscape (and wealth) of the community at that time, and a basis for assessing any post-COVID changes, which are still underway and too early to assess.

One feature captured in the data collection was the postal code of the location(s) where the organizations deliver their sport programming, enabling a geospatial information system (GIS) analysis of those points. GIS mapping can be used to profile the location and density of sport organizations in a community to better understand that landscape (cf. Twombly et al., 2000). The ArcGIS Online web-based platform (Environmental Systems Research Institue [ESRI], 2021) was used to map and analyze the delivery points identified for the 218 organizations, with multiple facilities or sites used by some organizations (e.g., a baseball or hockey club). Where no specific facility was used for program delivery (such as with a cycling or running club), the meeting point for the activity was reported. The data spreadsheet for the 218 organizations was extended to account for each delivery point, giving each location used by an organization its own point on the map, and allowing for a smooth upload to the ArcGIS platform. Through the platform one can map the population of data points in general, or by specific features of the organization at each point (e.g., nonprofit, shared facility, etc.). This allows for different maps highlighting the location and density of select organizational features of the community sport landscape. Following the upload to ArcGIS Online, the City of London boundaries of the five general planning districts (Northwest, Northeast, Central, Southwest, Southeast; City of London, 2019a) were overlayed on the map in order to ascertain sport delivery location and density by area.

To determine the profile of private community sport organizations in the City, data were analyzed descriptively (frequencies and percentages of organizations according to various features). In addition to a visual analysis of the sport delivery maps, point density was calculated to determine the concentration of sport delivery points in each of the five planning districts, dividing the number of points by the area (km^2^). To enhance the comparison of sport delivery density across the areas, it was of interest to align delivery density with population density. Thus, we calculated population density (popn/area) and assessed the correlation with delivery density across the sample. The population data and area of each planning district was obtained from the *2019 Parks and Recreation Master Plan* (City of London, 2019a) and the City of London Map Gallery (https://maps.london.ca/WebDocuments/MapGallery/MapGallery/Index), respectively.

## Results

The profile of the 218 private organizations identified as offering sport programming in London illustrates the community sporting landscape, presented according to features of the scope, operations, and location of the organizations (see Table 2).

**Table 2:** Profile of private community sport organizations in London, Ontario.

**Organizational Feature**	**Subgroup**	***n***	**%**
**Scope**			
Primary sport offered (*n* = 218 orgns)	Adventure sports (e.g., climbing, high ropes, mountain-biking, trampoline)	6	2.8
	Aquatics (e.g., swimming, scuba, diving, waterpolo)	10	4.5
	Archery	1	0.5
	Baseball	14	6.4
	Basketball	9	4.1
	Boating activities (e.g., rowing, dragon boat racing, kayaking, sailing)	3	1.3
	Bocce ball/Lawn bowling	3	1.3
	Bowling	2	0.9
	Cheer	2	0.9
	Contact sports (e.g., boxing, martial arts, karate, wrestling)	17	7.8
	Cricket	3	1.3
	Curling	1	0.5
	Cycling (road, track)	6	2.7
	Darts	2	0.9
	Dodgeball	1	0.5
	Equestrian and Horseback riding	1	0.5
	Fencing	1	0.5
	Field hockey	1	0.5
	Fishing	2	0.9
	Football	5	2.3
	Golf	17	7.8
	Gymnastics (artistic, rhythmic)	7	3.2
	Hockey (also ball hockey, roller hockey, ringette – 1 each)	24	11
	Ice skating	2	0.9
	Lacrosse	3	1.4
	Paintball & Airsoft	1	0.5
	Para sports, Special Olympics etc.	5	2.3
	Racket sports (e.g., tennis, badminton, pickleball)	7	3.2
	Roller derby	2	0.9
	Rugby	1	0.5
	Running, Cross-country, Speed walking	4	1.8
	Snow sports (Alpine skiing, Nordic Skiing, Snowboarding)	2	0.9
	Soccer	20	9.2
	Softball	3	1.4
	Speed skating	1	0.5
	Track and field	2	0.9
	Triathlon	2	0.9
	Ultimate	1	0.5
	Volleyball	5	2.2
	Weightlifting	1	0.5
	Multi-sport	18	8.2
Multiple sports offered	Yes	63	28.9
(*n* = 218)	No	155	71.1
Program age target	Children (<10 years)	53	28.6^a^
(*n* = 185)	Youth (10–17 years)	65	35.1
	Adults (18–54 years)	40	21.6
	Seniors (55+ years)	10	5.4
	All ages	100	54
Other offerings (social)	Yes	121	55.7
(*n* = 218)	No	97	44.3
**Operations**			
Sector	Commercial	92	42.4
(*n* = 217)	Nonprofit	125	57.6
Program type	Open	10	4.6
(*n* = 217)	Closed	151	69.6
	Open and closed	56	25.8
	Commercial, open	9	4.1 (9.8%)^b^
	Commercial, closed	45	20.7 (48.9%)^b^
	Commercial, open and closed	38	17.5 (41.3%)^b^
	Nonprofit, open	1	0.5 (0.8%)^c^
	Nonprofit, Closed	106	48.8 (84.8%)^c^
	Nonprofit, Open and Closed	18	8.3 (14.4%)^c^
Independent/ Affiliated/	Independent	173	79.3
Franchise status (*n* = 218)	Affiliated	28	12.8
	Franchise	17	7.8
Facility use	Shared	115	54.2
(*n* = 212)	Exclusive	85	40.1
	No Facility	12	5.6

a *Percentages total more than 100 as some organizations serve more than one age group (organizations that serve more than two age groups are considered to serve “all ages” and are counted once)*.

b *Proportion of commercial sport organizations*.

c *Proportion of nonprofit sport organizations*.

### Scope of Sport Organizations

#### Sports Offered

Forty-two different sports (or groups of similar sports; e.g., boating, contact sports) constitute the primary offering of the 218 organizations. The most common sport is ice hockey (11% of organizations), followed by soccer (9.2%), multi-sport (8.2%), and contact sports and golf (both 7.8%). Just over one-quarter of organizations (28.9%) offer other sports in addition to their primary offering; for example, a gymnastics club that also offers cheerleading.

#### Program Age Targets

Organizations were counted according to whether they offer programming to children (<10 years of age), youth (10–17 years), adults (18–54 years) or seniors (55 years and older), or some combination. This information was available publicly for 85% of the sample. About half (54%) of the organizations serve participants of all ages (and were counted once). Of the remaining clubs, one-third (35.1%) offer programming for youth and about one-quarter (28.6%) serve children. One-fifth (21.6%) provide programs for adults, and as few as 5.4% of the organizations offer programming specifically for seniors. Overall, it can be determined that most organizations serve youth (89.1%), followed by children (82.6%), adults (75.6%), and to a far lesser extent seniors (59.4%).

#### Other Offerings

Just over half (55.7%) of all the organizations offer social opportunities and programs in addition to their sport programs. These options range from birthday parties and corporate events that can be booked by anyone, to post-sporting activity meals and parties for members.

### Operations

#### Sector and Program Type

Over half (57.6%) of the community sport organizations operate in the nonprofit sector, with the remainder (42.4%) operating on a commercial or for-profit basis. The organizations were further categorized as offering closed programming to individuals who pay an extended membership to participate (total 69.6%), open drop-in opportunities with a one-time financial commitment (4.6%), or both (25.8%). Further analysis revealed the large majority of the nonprofit organizations (84.8%) offer only closed programming. Almost half of the commercial organizations (48.9%) also offer only closed programming, however 41.3% offer both open and closed options. Very few nonprofit organizations offer both (14.4%), and very few organizations in either sector offer exclusively open or drop-in programs.

#### Independent/Affiliated/Franchise Status

The large majority (79.3%) of organizations operate independently, with no apparent formal linkages to, and without the governance or legal oversight of, a parent or umbrella organization. Of the remainder, a small proportion (12.8%) are affiliated with an umbrella organization that directly supports their operation (e.g., a minor hockey league or alliance), or are a franchise operation of a larger corporation (7.8%, e.g., Sky Zone trampoline park, Premier Martial Arts London).

#### Facility Use

Over half the organizations (54.2%) rely on the use of shared facilities for their programming, including pools, fields, and school gyms. The remainder have exclusive access to their facility (40.1%) or rely on no formal facility at all (5.6%, e.g., a cycling club).

### Location

**Figure 1:**
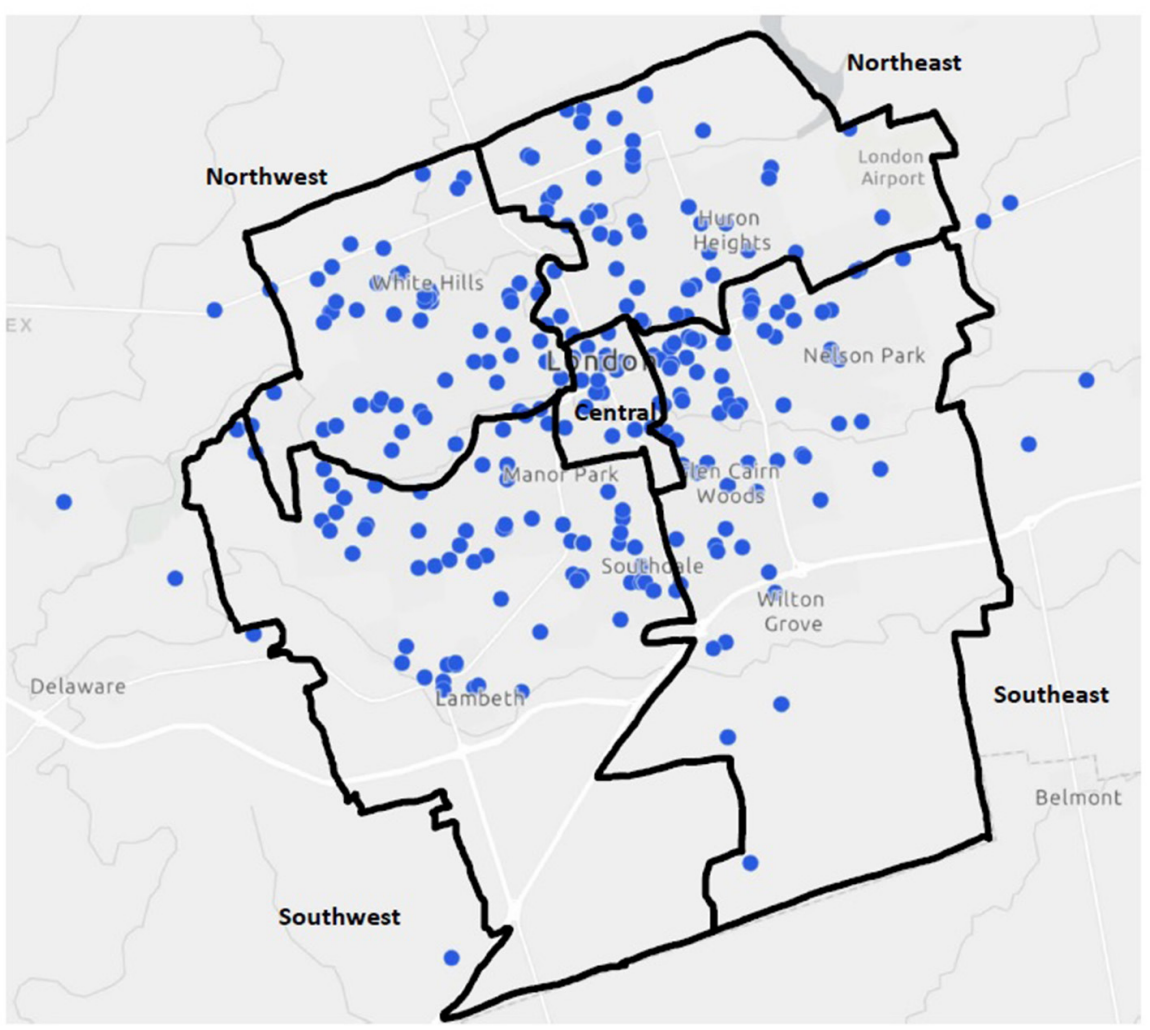
All sport delivery points in the five City of London planning districts.

**Figure 2:**
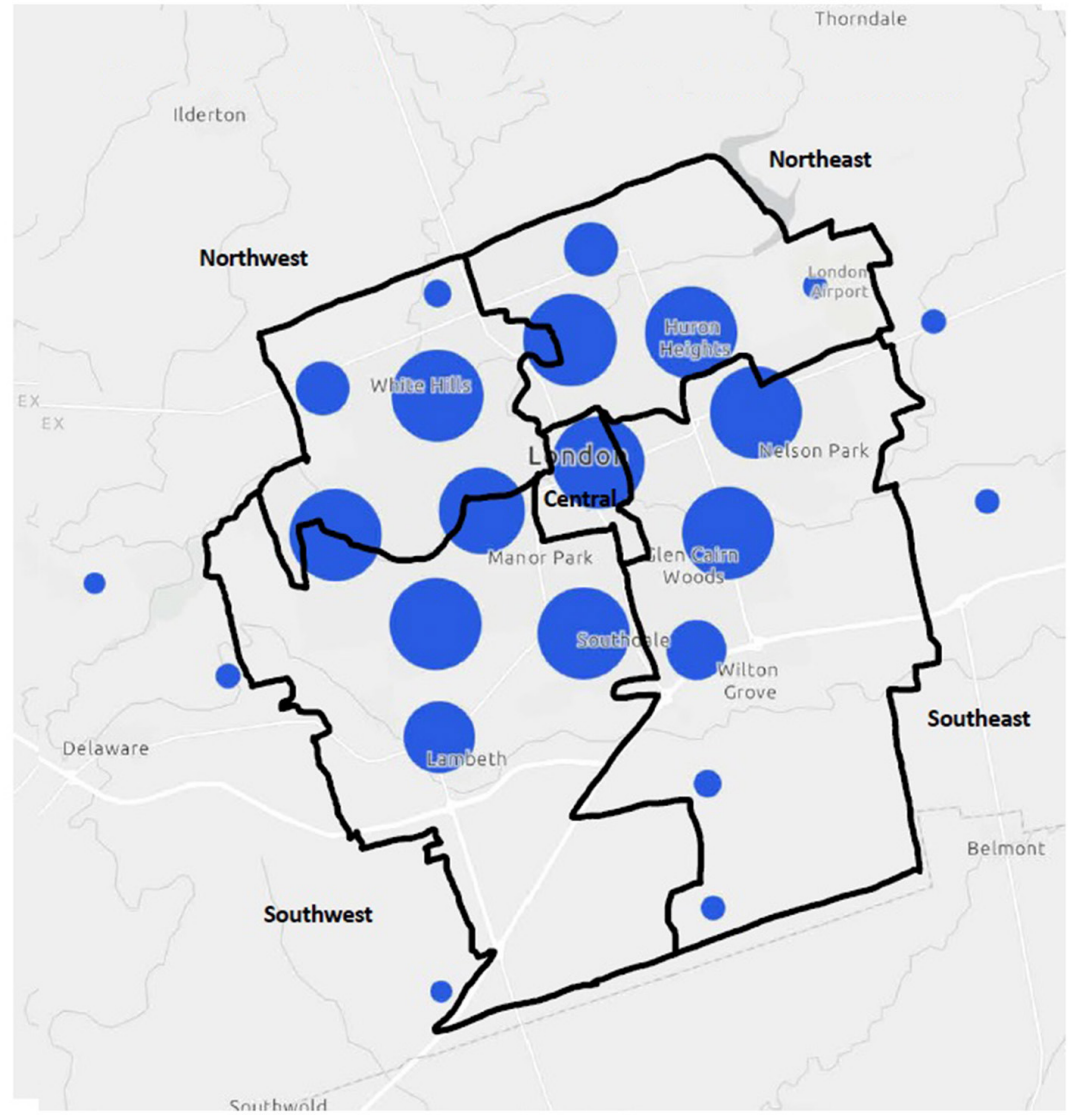
Clustered sport delivery points in the five City of London planning districts.

Geospatial mapping revealed that program delivery by private community sport organizations can be found in all five general planning districts of the City of London (see Figure 1). Of note, several delivery points for London-based community sport organizations lie outside the City boundaries. Despite the spread of delivery across the City, some differences in sport delivery density among the districts are apparent (see Table 3). By area size in square kilometers, sport delivery density is highest in the Central area (2.54 points/km^2^), followed by the Northwest (1.75 points/km^2^) and Northeast (1.48 points/km^2^), and the far less dense Southeast (0.88 points/km^2^), and Northeast (0.87 points/km^2^) districts. The delivery of sport by private community organizations is thus much more concentrated in the core of the City, where there is also greater population density (see Table 3). This possible alignment was examined with a Pearson correlation analysis with the sample, which revealed a significant and direct association between population density and sport delivery density (*r* = 0.89, *p* < 0.05), with population per district size corresponding with number of delivery points per district size for the sample. Further proportional symbol mapping (Figure 2) illustrates the clustering or spread of the delivery points across a district. It appears there are larger and smaller clusters of delivery points in each district (with the exception of the smaller Central area), although the clusters are less spread out in the Southeast and Southwest districts. The concentration of delivery points in the northern areas of the southern districts is nonetheless consistent with the population distribution in those areas.

**Table 3:** City of London planning areas profile and sport delivery density.

**General area features**	**Northwest**	**Northeast**	**Central**	**Southeast**	**Southwest**
Population	86,900	77,700	72,400	70,900	101,100
Area (km^2^)	60	67.1	9.45	143.8	140.3
Sport delivery points (*N* = 473)	105	98	24	126	121
Population density (popn/km^2^)	1448	1158	7661	493	721
Sport delivery density (# points/km^2^)	1.75	1.48	2.54	0.88	0.87

## Discussion

The landscape of community sport offered by private nonprofit and commercial organizations in the City of London is characterized by a wide variety of sports catering to different interests. In addition to the 42 sports (or groups of sports) offered, multi-sport organizations constitute one of the largest proportions of organizations, and over a quarter offer at least one other sport (e.g., golf plus curling). It is not surprising that the largest number of organizations offer ice hockey and soccer, as those are consistently two of the most popular sports by overall population in Canada (Statistics Canada, 2019), and especially for children and youth [Canadian Fitness Lifestyle Research Institute [CFLRI], 2013], which are the cohorts served by the largest proportion of community sport organizations overall. However, there is also at least one organization supporting what may be considered newer and alternative sports in the community, such as cricket, paintball and airsoft, and roller derby. The community appears to be well-served by the breadth of sporting activities available, with at least some opportunity for inclusion and engagement in the sport of one's choice, and the expression and further development of one's identity in and through the sport and organization (Ulseth, 2004).

It is notable, however, that relatively fewer organizations offer programming for seniors, either exclusively or in combination with serving other age groups. While this may be proportional with the population demographics of London, it may be a point of concern for the City, which has sport and recreation programming for an aging population on its radar and which may not be well-supported by private sport organizations. Anticipated demographic shifts (City of London, 2019a) have implications for both the need and opportunity for community development through expanded sport opportunities for seniors through private organizations. Nonetheless, half of the organizations offer programming for all age groups, promoting multi-generational participation and engagement opportunities in a variety of community-based activities.

In addition to sport, over half of the organizations provide social offerings. While some of these are linked directly to member participation (post-game parties), other organizations do make themselves available for personal and corporate events where Londoners can enjoy a given sport (or multiple sports) in the context of a social event. This extends the opportunity for individuals to connect with a club and sport that is already serving others in the community. This aspect of the organizations profile suggests there is room in the community sport landscape to further develop this opportunity for even broader social reproduction (DeFilippis and Saegert, 2012).

This wide variety of sports offered and range of ages targeted is, nonetheless, within the nonprofit sector or the commercial sector which can have implications for access and engagement. About half the private sport organizations in London operate as nonprofit entities, and so are more likely to offer programs at lower prices, because of their focus on member needs and reliance on voluntary work (Hallmann et al., 2015; Rossi et al., 2020). As nonprofits they are also more likely to *have* opportunities for engagement because of their focus on democratic and participatory decision making by members (Hallmann et al., 2015; Rossi et al., 2020). There is an opportunity for active citizenship and sociopolitical development in nonprofit sport organizations as individuals are able to become involved in the sustainability of the organization and the sport (Cousens and Barnes, 2009; Wheaton et al., 2017). This may also have implications for more extensive social capital in the nonprofit setting (Hallmann et al., 2015; Hill et al., 2021), including relationships and networks that ‘bridge’ a wider variety of individuals with active involvement in the organization (Doherty and Misener, 2008). Commercial sport organizations, in contrast, which make up just less than half the private institutions in the City, are characterized by a profit orientation, and customers (rather than members; Ulseth, 2004) who typically do not have an opportunity to engage in organizational decision making (Lang et al., 2019; Rossi et al., 2020). While further research can provide insight to implications of nonprofit vs. commercial organizations for access and engagement, and further social capital, the apparent balanced proportions of these organizations is an important element of the sport landscape in this community. The current study was cross-sectional and there is not adequate corresponding data to examine whether there has been a shift in the proportion of nonprofit and commercial organizations in the City over time. However, Rossi et al. (2020) describe a trend of increasing numbers of commercial sport organizations, which should be monitored in this community as well.

The profile generated in this study further highlights that closed programming—available to customers or members only with a typically extended registration and accompanying financial commitment, with implications for access—is found as the only option in almost 70% of the organizations. Interestingly, closed programming is far less likely to be found in commercial organizations (20.7%) compared to non-profits (48.8%), although it is the most common option in both. It is not surprising that the large majority of non-profit sport organizations focus on closed programming, as they rely heavily on sustained revenues from membership fees as their main source of income (Doherty et al., 2014). Stenling (2013) also notes that nonprofit clubs are particularly challenged to offer open, pay-as-you-go, or ‘drive-in sport’ options, even though they may be well-suited to do so, because it contradicts their organizational identity. One-quarter of organizations in London offer both open and closed options, however these too are much more likely to be found with commercial programs, where fees are likely to be relatively higher. Open programs may be more accessible and thus socially inclusive to some marginalized groups, and certainly more flexible, because they do not rely on an extended financial commitment, however they are offered by less than a third of the private sport organizations in this community. Thus, the community sport landscape in London is characterized by a heavy focus on closed or member/registration-based programming that may limit access and thus participation and engagement, yet it is found predominantly in nonprofit organizations that likely have more reasonable fees that may offset access constraints. Open program options are more likely to be found in the commercial sector, where pay-as-you-go may promote access and participation, although higher fees that can be expected in the profit-oriented organizations may offset accessibility.

The landscape of community sport organizations in London is also characterized by their independent, affiliated or franchise status, which may distinguish those (independent) which are more bottom-up, community-driven organizations (Pedlar, 1996; Bolton et al., 2008) from those (affiliated, franchise) that are guided or even established from the top-down or beyond the community by umbrella or parent organizations. As independent entities, almost 80% of sport clubs in London may be considered testaments to community development, as institutions where collective social reproduction, that sustains a community and its members (Vail, 2007; DeFilippis and Saegert, 2012) is paramount. Working together, in either paid or voluntary work in commercial or nonprofit organizations, Londoners themselves have established, and sustain, most of the sport organizations in the community. The community-driven trust and social capital that is likely built within and beyond those organizations (Doherty and Misener, 2008), and the local identity and pride they may engender (Misener and Mason, 2006), is a valuable aspect of the landscape. Social capital may also be particular to organizations that share facilities for their programming, given the need for partnerships and cooperation to ensure a successful system (Misener and Doherty, 2012). Those facilities are often in the municipal and public school domains, and further indicative of the reach of community sport organization networks (Doherty and Misener, 2008). Over half of the community sport organizations in London rely on shared facilities, and while that is noted as a constraint to programming (Doherty et al., 2014), an upside may be the community development that is generated in that sharing (cf. Rosentraub and Ijla, 2008).

Sport is delivered by private community organizations in all five general planning districts of the City, and the density of the delivery points corresponds directly with the population density of the districts. However, the maps reveal that sport delivery is generally clustered in one or two areas within each district, rather than spread out. This can have implications for ease of access (Ulseth, 2004), for example, depending on public transit availability within a district and to/from the cluster of delivery points. There can also be implications for population growth within a district and proximity to the current clustered delivery points. This may be an issue particularly in a larger area like the Southwest district, which has the greatest anticipated population growth in the next 20 years (City of London, 2019a).

### Contribution and Implications for Theory and Practice

The community sport landscape highlighted in this study provides a foundation for considering private organizations' contribution to aspects of community development, and the asset-based planning, implementation, and evaluation that are fundamental to the betterment of a community (Vail, 2007; Schulenkorf, 2012). It provides a benchmark for City of London planners to be aware of the highlighted gaps, and opportunities, in the sport landscape; recognizing that the municipality may already be filling such gaps and addressing such opportunities, with its mandate to “build a better London for all” (City of London, 2019b, p. 5). The highlighted gaps and opportunities include the potential for more open programming that may promote accessibility and social inclusion, a possible gap in seniors' programming in private community sport delivery, and implications for the density and spread of sport delivery by private organizations as the population of districts grow. The landscape also has implications for private community sport organizations that should be aware of some of those same gaps and opportunities: the potential for more open programming, more seniors' programming, and access to sport delivery points as the population of London continues to grow, particularly in the peripheral districts.

Thus, the landscape exercise in general provides a platform, and prompt, for reflection on the possible implications for community development in terms of social inclusion, citizen engagement, individual and community identity, and social capital that may be engendered through community sport organization programs and opportunities. It also provides a benchmark to inform municipal policy, strategy, and planning for sport programming in a community. There appears to be value in continuing to develop the profile of sport in this community, to better understand the landscape of organizations and program delivery, and their implications for community development.

The study also contributes more broadly to the slowly growing body of research taking stock of small community-based organizations (cf. Gronbjerg and Nelson, 1998; Twombly et al., 2000; Toepler, 2003; Elson and Hall, 2012). While we did not measure community development directly, the conceptual framing for this study brings into focus the possible alignment between features of local organizations and their possible implications for the betterment of a community (cf. Doherty and Rich, 2015).

## Conclusion

### Limitations

A number of limitations must be outlined, as they characterize the context of our study and provide a springboard for future research. We relied on data about the population of private community sport organizations that was available only in the public domain. Some of the information may have been out of date, and some of it was not available at all, thus rendering our database incomplete. These limitations may have compromised our findings, however they provide another important piece of information about the landscape of these organizations: what information do they make available publicly to the community? Further, the cross-sectional design of the study meant we captured the landscape at only one point in time, although a strength of the study design is that the data from all organizations were collected within a narrow and thus common time frame for all. The study was delimited to private sport organizations, yet public sport programs are a critical part of the full sport offerings in communities. The landscape derived here is, thus, inherently limited without this data, prompting continued investigation.

An important limitation of our study is the presumption that sport is inherently good, although of course there can be negative practices and consequences, such as social exclusion, exclusionary social capital, and so on (Doherty and Rich, 2015). It is important to acknowledge that sport is not always ‘for good’ (cf. Maxwell et al., 2013; Wheaton et al., 2017), and future research may consider aspects of the community sport landscape that do not serve the best interests of members of a community, and even present negative circumstances.

### Future Research

Our beginning efforts to map the landscape of private community sport organizations in one community—focused on scope, operations and location—has great potential for further investigation, as well as extension to other communities. The landscape presented here can be extended with the consideration of other organizational features that we captured in the broader project but did not consider in the current study (see Appendix). Both theoretical and practical interests should guide the selection of features, perhaps building to the development of a taxonomy of types of organizations (cf. Nichols et al., 2015; Lang et al., 2019). An important step will be to confirm, update and extend the profile through primary data collection with each organization. It may be most interesting to do this at a critical post-COVID point where sport organizations have returned to sustained program delivery. Data at both (or multiple) time points will facilitate a longitudinal examination of the sporting landscape. New features may also be included, such as organization size in terms of number of members, as an indicator of capacity for social impact. These data are not available on organizations' websites and would have to be collected directly. New features that provide a “critical [communitarian] perspective of community” (Rich et al., 2021, p. 7), such as organizations' attention to social justice issues, may be of particular interest in the consideration of sport for community development. Further, some features considered here may be extended to additional insights, such as details about facilities access and cost, as critical aspects of organization operations and programming (Doherty et al., 2014). Future research, and the landscape, should also capture public sport offerings for a comprehensive picture of the potential for community development, and direction for municipal sport policy, strategy, and planning.

The current project and findings also provide a springboard to further explore and build on the limited research to date comparing nonprofit and commercial sport clubs. There may be sector blurring (Misener and Misener, 2017) such that scope and operation features are indistinguishable as nonprofit and commercial organizations increasingly “operate in similar ways and markets” (Rossi et al., 2020, p. 738; also Enjolras, 2002; Hallmann et al., 2015; Lang et al., 2019). The wealth of a community depends (in part) on the health of the private organizations that provide community sport offerings, and thus future research should consider the implications of nonprofit/commercial competition, sustainability, and capacity for growth that can support community development.

## Data Availability Statement

The original contributions presented in the study are included in the article/supplementary materials, further inquiries can be directed to the corresponding authors.

## Author Contributions

This student-based project was led by AD. SP and JR contributed to the conceptual framing and writing. AP and KS completed the data collection, and AP conducted the geospatial analysis. All authors contributed to the article and approved the submitted version.

## Funding

This study was supported in part by a MITACS Research Training Award (IT20786).

## Conflict of Interest

The authors declare that the research was conducted in the absence of any commercial or financial relationships that could be construed as a potential conflict of interest.

## Publisher's Note

All claims expressed in this article are solely those of the authors and do not necessarily represent those of their affiliated organizations, or those of the publisher, the editors and the reviewers. Any product that may be evaluated in this article, or claim that may be made by its manufacturer, is not guaranteed or endorsed by the publisher.

## Appendix

**Full list of private community sport organization features**

Name

Location

Sector (nonprofit, commercial)

Independent, affiliated, franchise

Accreditation with governing body

History (years)

Symbol/logo

Mission statement

Goals, purpose

Facility use (shared, exclusive, no facility)

Facility features

Sport(s) offered

Programs offered

Program type (open, closed, both)

Pricing

Other offerings

Number of participants

Participants' label (member, customer, etc.)

Program age target (children, youth, adults, seniors, all ages)

Coach, officials accreditation

Policies, procedures (number, type)

Paid, voluntary personnel (yes/no, number)

Social media presence (platforms)

Technology use (esp. contemporary)

Connection(s) with other organizations (yes/no, who)

Social responsibility, charity activities

Environmental conscientiousness (yes/no, what)

Health promotion focus (yes/no, what)

Safety, risk management
